# Role of DNA Methylation and CpG Sites in the Viral Telomerase RNA Promoter during Gallid Herpesvirus 2 Pathogenesis

**DOI:** 10.1128/JVI.01488-20

**Published:** 2020-11-09

**Authors:** Srđan Pejaković, André Claude Mbouombouo Mfossa, Laëtitia Wiggers, Ahmed Kheimar, Damien Coupeau, Benedikt B. Kaufer, Benoît Muylkens

**Affiliations:** a Namur Research Institute for Life Sciences, University of Namur, Namur, Belgium; b Department of Veterinary Medicine, University of Namur, Namur, Belgium; c Institut für Virologie, Freie Universität Berlin, Berlin, Germany; d Department of Poultry Diseases, Faculty of Veterinary Medicine, Sohag University, Sohag, Egypt; University of Arizona

**Keywords:** Gallid herpesvirus type 2, viral telomerase RNA subunit, c-Myc, E-box, epigenetic regulation, DNA methylation, telomerase activity, virus-induced oncogenesis

## Abstract

Gallid herpesvirus type 2 (GaHV-2) is an oncogenic alphaherpesvirus that induces malignant T-cell lymphoma in chicken. GaHV-2 encodes a viral telomerase RNA subunit (vTR) that plays a crucial role in virus-induced tumorigenesis, enhances telomerase activity, and possesses functions independent of the telomerase complex. vTR is driven by a robust viral promoter, highly expressed in virus-infected cells, and regulated by two c-Myc response elements (c-Myc REs). The regulatory mechanisms involved in controlling vTR and other genes during viral replication and latency remain poorly understood but are crucial to understanding this oncogenic herpesvirus. Therefore, we investigated DNA methylation patterns of CpG dinucleotides found in the vTR promoter and measured the impact of methylation on telomerase activity. We demonstrated that telomerase activity was considerably increased following viral reactivation. Furthermore, CpG sites within c-Myc REs showed specific changes in methylation after *in vitro* reactivation and in infected animals over time. Promoter reporter assays indicated that one of the c-Myc REs is involved in regulating vTR transcription, and that methylation strongly influenced vTR promoter activity. To study the importance of the CpG sites found in c-Myc REs in virus-induced tumorigenesis, we generated recombinant virus containing mutations in CpG sites of c-Myc REs together with the revertant virus by two-step Red-mediated mutagenesis. Introduced mutations in the vTR promoter did not affect the replication properties of the recombinant viruses *in vitro*. In contrast, replication of the mutant virus in infected chickens was severely impaired, and tumor formation completely abrogated. Our data provides an in-depth characterization of c-Myc oncoprotein REs and the involvement of DNA methylation in transcriptional regulation of vTR.

**IMPORTANCE** Previous studies demonstrated that telomerase RNAs possess functions that promote tumor development independent of the telomerase complex. vTR is a herpesvirus-encoded telomerase RNA subunit that plays a crucial role in virus-induced tumorigenesis and is expressed by a robust viral promoter that is highly regulated by the c-Myc oncoprotein binding to the E-boxes. Here, we demonstrated that the DNA methylation patterns in the functional c-Myc response elements of the vTR promoter change upon reactivation from latency, and that demethylation strongly increases telomerase activity in virus-infected cells. Moreover, the introduction of mutation in the CpG dinucleotides of the c-Myc binding sites resulted in decreased vTR expression and complete abrogation of tumor formation. Our study provides further confirmation of the involvement of specific DNA methylation patterns in the regulation of vTR expression and vTR importance for virus-induced tumorigenesis.

## Abstract (precis)

Previous studies demonstrated that telomerase RNAs possess functions that promote tumor development independent of the telomerase complex. vTR is a herpesvirus-encoded telomerase RNA subunit that plays a crucial role in virus-induced tumorigenesis and is expressed by a robust viral promoter that is highly regulated by the c-Myc oncoprotein binding to the E-boxes. Here, we demonstrated that the DNA methylation patterns in the functional c-Myc response elements of the vTR promoter change upon reactivation from latency, and that demethylation strongly increases telomerase activity in virus-infected cells. Moreover, the introduction of mutation in the CpG dinucleotides of the c-Myc binding sites resulted in decreased vTR expression and complete abrogation of tumor formation. Our study provides further confirmation of the involvement of specific DNA methylation patterns in the regulation of vTR expression and vTR importance for virus-induced tumorigenesis.

## INTRODUCTION

Gallid herpesvirus 2 (GaHV-2) is an avian alphaherpesvirus that causes highly malignant T-cell lymphoma considered to be the most prevalent cancer in the animal kingdom (1, 2). In susceptible chickens, the ultimate consequence of the host-virus interactions is the transformation of the CD4^+^ T cells (2), which eventually proliferate to form visceral lymphomas, causing high mortality (3). GaHV-2 belongs to the genus *Mardivirus*, into which two other closely related but distinct species have been grouped, represented by Gallid herpesvirus type 3 (GaHV-3) and Meleagrid herpesvirus type 1 (MeHV-1). Only GaHV-2 causes clinical disease in chickens, while the other two species are nonpathogenic (4). The GaHV-2 genome belongs to the class E genome with a size of 175 to 180 kbp. The GaHV-2 genome consists of a unique long (U_L_) and a unique short (U_S_) segment bracketed by inverted repeats known as terminal and internal repeats long (TR_L_ and IR_L_) and terminal and internal repeats short (TR_S_ and IR_S_) (2). GaHV-2 genes, similar to those of other herpesviruses, also belong to three kinetic classes of immediate early, early, and late genes based on the requirements for viral protein synthesis and DNA replication (5). During GaHV-2 infection, several viral factors, proteins, and diverse RNAs, including the major oncoprotein Meq (6), viral interleukin-8 (7), and GaHV-2-encoded microRNAs (miRNAs), contribute to lymphomagenesis (6–8). In addition, GaHV-2 encodes two copies of the viral telomerase RNA subunit (vTR), which is expressed both during productive infection and in virus-transformed T cells. vTR is a noncoding RNA that shares 88% sequence homology with chicken telomerase RNA subunit (chTR), and it was likely acquired from the chicken genome during virus-host coevolution. vTR interacts with the chicken telomerase reverse transcriptase subunit (TERT) for enhancing telomerase activity and contributing to the efficient and rapid onset of lymphoma (9–11). Furthermore, vTR relocalizes ribosomal protein L22 that plays an essential role in T-cell development and transformation (12). vTR functions independent of the telomerase complex are also responsible for tumor progression and dissemination (10, 11). It is the most abundant viral transcript detected in GaHV-2-induced tumor cells and is much more highly expressed than chTR in infected cells, likely due to differences in their promoters. It has been shown that the tumor incidence was severely impaired in chickens infected with GaHV-2 mutants harboring the chTR promoter instead of the native vTR promoter within the virus genome, confirming the vTR promoter plays an important role in vTR functions (13). The vTR promoter has additional AP-1 sites, c-Myc transcription factor binding sites (namely, E-box 1, E-box 2, and E-box 3), and EBS transcription factor binding sites. However, it was demonstrated that E-box 1 was not functional (14). It has been shown that the c-Myc oncoprotein is involved in the regulation of vTR during GaHV-2-induced lymphomagenesis (14) and that increased expression of vTR is essential for the oncogenic potential of the virus (13). During the viral life cycle, transcriptional modifications and epigenetic changes, together with posttranscriptional and posttranslational modifications, regulate expression of cellular and viral genes. Altogether, they allow GaHV-2 to switch between the productive and latent phase, and to induce cellular transformation (15). This study aimed to investigate the epigenetic mechanisms involved in the switch between the productive and latent phase of GaHV-2 infection. We established 5-methylcytosine (5mC) patterns for the vTR promoter *in vitro* and *in vivo* and determined the impact of methylation on the telomerase activity and c-Myc response elements of the vTR promoter. Furthermore, to study the importance of the c-Myc binding sites in virus-induced tumorigenesis, a recombinant virus bearing mutations in functional-Myc response elements (c-Myc REs), as well as revertant virus, were generated using the bacterial artificial chromosome of a highly oncogenic GaHV-2 strain (pRB-1BΔIRL) by two-step Red-mediated mutagenesis (16). Susceptible (B^13^B^13^) chickens were infected with the recombinant viruses to assess the impact of c-Myc RE mutations. To investigate GaHV-2 replication and telomerase activity during the course of infection, blood and feather follicle epithelium (FFE) were collected at specific time points from infected chickens. Animals were daily monitored for the development of clinical symptoms and euthanized at 55 days postinfection, to assess the number of tumors developed in visceral organs.

## RESULTS

### Impact of DNA methylation on the telomerase activity and changes of vTR promoter DNA methylation patterns.

**FIG 1:**
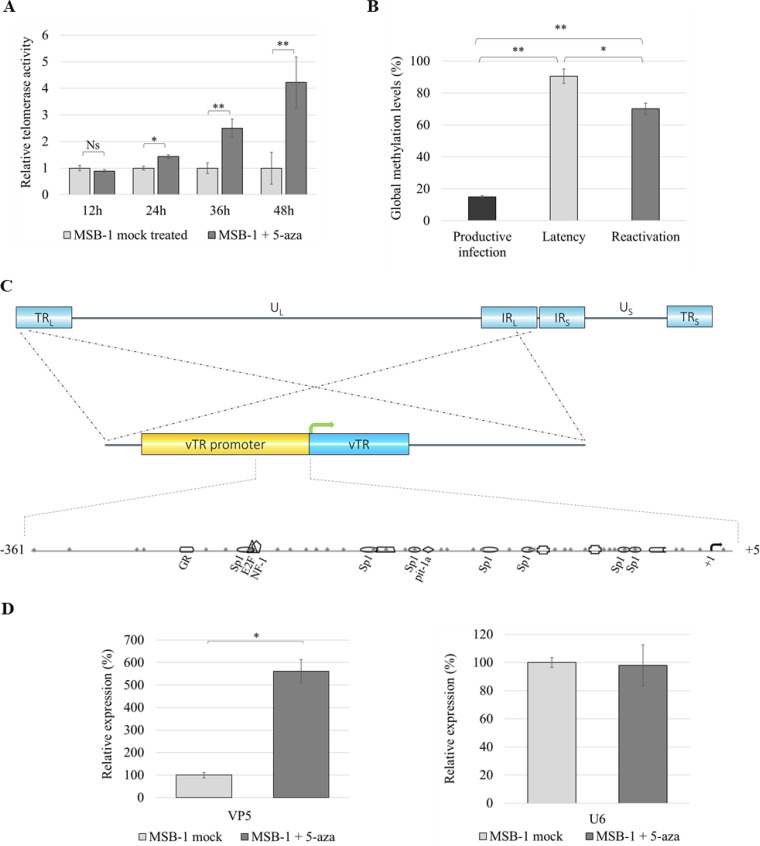
Effect of demethylation agent on relative telomerase activity, global methylation levels of the vTR promoter, and reactivation rate. (A) Induction of viral reactivation in the MSB-1 cell line with 5-azacytidine (5-aza), a DNA methyltransferase inhibitor, at specific time points during the period of 48 h. A significant increase in telomerase activity during the 48 h after the treatment was observed. (B) Using the bisulfite genomic sequencing assay, significantly higher 5mC levels were found *in vitro* during latency, compared to productive infection. Following induction of the reactivation, methylation levels significantly dropped to 70%. The determination of the significant difference was performed on 50 randomly picked bacterial colonies using Student’s *t* test, for which *P* ≤ 0.05 was considered statistically significant. (C) Gallid herpesvirus type 2 (GaHV-2) genome consists of unique long (U_L_) and unique short (U_S_) regions flanked by the long terminal (TR_L_) and internal (IR_L_) repeats, and terminal (TR_S_) and internal short repeats (IR_S_), with the two copies of vTR located within in the TR_L_ and IR_L_ regions. Shown is a schematic representation of the studied region of the vTR promoter with 38 CpG positions and specific response elements obtained with Genomatix analysis software. The black arrow represents the transcription start site (TSS). (D) Relative expression of VP5 gene was obtained using the Livak method and is shown relative to the cellular U6 control gene in MSB-1 cells (mock) treated with 5-azacytidine. Student’s *t* test: ns, not significant; *, *P* ≤ 0.05; **, *P* ≤ 0.005.

To study the impact of DNA methylation on telomerase activity in the lymphoblastoid cell line (MSB-1) latently infected and transformed by GaHV-2, a telomeric repeat amplification protocol (TRAP) assay was performed. Relative telomerase activity was measured and compared between mock-treated, and 5-azacytidine-treated cells at specific time points, as shown in Fig. 1A. The results showed a slight increase in telomerase activity during the first 24 h after treatment with the demethylating agent, and notably more potent activity after 48 h of exposure to the demethylating agent (Fig. 1A). In addition, significantly higher relative expression of the gene encoding the major viral capsid protein VP5, involved in a productive phase of the viral life cycle, confirmed that demethylation using 5-azacytidine induces viral reactivation from latency (Fig. 1D).

**FIG 2:**
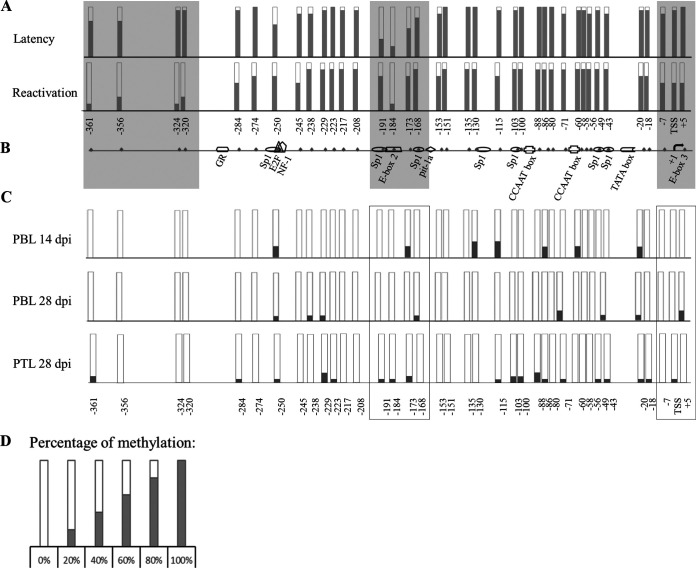
Methylation profiles of the vTR promoter obtained by bisulfite genomic sequencing assay. (A) Detailed methylation mapping showing different CpG methylation patterns in latency and after viral reactivation *in vitro*, in areas upstream of the transcription start site (TSS) and functional c-Myc response elements (c-Myc REs) in the vTR promoter. Gray shaded areas represent sections of the vTR promoter, where significant changes of methylation patterns were observed. (B) Schematic representation of the vTR promoter with CpG positions and specific response elements obtained with Genomatix analysis software. The black arrow represents the transcription start site (TSS). Each CpG position is represented with a white bar numbered with respect to the TSS. (C) For the *in vivo* samples, DNA from peripheral blood leukocytes (PBLs) was isolated at days 14 and 28 postinfection, and total peripheral tumor leukocytes (PTLs) were isolated at day 28 postinfection. Significantly lower global methylation levels compared to *in vitro* samples were detected, with specific changes in methylation patterns surrounding the areas (in boxes) of c-Myc REs in the vTR promoter; however, the changes were not significant. (D) Legend for methylation percentages for the *in vitro* and *in vivo* samples. Gray color in bars represents the percentage of methylation for a single CpG position calculated as the rate of isolated cytosines effectively converted into thymine after bisulfite treatment. The determination of significant differences was performed on 50 randomly selected bacterial clones using the Student’s *t* test.

With the aim to explore the potential role of DNA methylation in the control of vTR expression, the DNA methylation landscape of the vTR promoter was characterized at the key steps of viral infection. CpG methylation patterns on the vTR promoter (Fig. 1C), stretching from 5 nucleotides (nt) downstream to 361 nt upstream of the transcription start site (TSS) (+1) were mapped using the bisulfite genomic sequencing assay in cell lines representing the productive or latent phase of the viral life cycle. Our results showed that total methylation on the vTR promoter was close to 15% during the productive phase in chicken embryo fibroblasts (CEF) infected with the highly virulent GaHV-2 RB-1B strain (Fig. 1B). Interestingly, the levels of CpG methylation throughout the vTR promoter in the quiescent virus genome in MSB-1 cells were high (up to 90%), which, upon viral reactivation, dropped down to 70% (Fig. 1B) with specific pattern changes (Fig. 2). This represents the first report on changes in DNA methylation levels in the vTR promoter measured at the relevant steps of GaHV-2 life cycle *in vitro*. Furthermore, our approach (as shown in Fig. 1D) using mock-treated MSB-1 cells harboring a quiescent GaHV-2 genome showed low VP5 expression, which changed after treatment with 5-azacytidine. This result indicated that MSB-1 cells represent a specific model for studying viral latency and reactivation in *in vitro* cultures.

To provide a more detailed view of DNA methylation, 38 CpG sites were mapped, together with the response elements within the vTR promoter, both *in vitro* and *in vivo*. Looking into specific methylation positions on the vTR promoter *in vitro*, CpG dinucleotides positioned in the transcription start site (TSS) and neighboring c-Myc RE (E-box 3) demonstrated a significant decrease in methylation, seen also in the area at the upstream end of the vTR promoter. Furthermore, CpG sites surrounding the second c-Myc RE (E-box 2) showed an increase in methylation compared to the latent state. For the rest of the vTR promoter, CpG methylation levels after reactivation were lower than during latency, however, without significant change (Fig. 2A). For the *in vivo* samples obtained from GaHV-2-infected chickens, overall global methylation levels were significantly lower compared to *in vitro* analysis (Fig. 2C). DNA methylation patterns on the vTR promoter obtained from peripheral blood leukocytes (PBLs) at 14 days postinfection (dpi) showed a decrease in methylation in the CpG site of the c-Myc RE (positioned two nucleotides downstream of TSS) compared to PBL samples collected at 28 dpi (Fig. 2C).

Moreover, for peripheral leukocytes isolated from tumor tissue (PTLs) at 28 dpi, methylation of CpG sites in both the TSS and the upstream c-Myc RE (E-box 2) was recorded (Fig. 2C). The changes in CpG methylation levels of the vTR promoter observed at specified time points *in vivo* were not significant; however, the changes in methylation status of CpG dinucleotides present in the functional c-Myc REs during the GaHV-2 life cycle were similar to the results obtained *in vitro*. In addition, the visible tendency for methylation increases at positions 88 to 103 in the peripheral tumor leukocytes (PTLs) at 28 dpi could implicate the importance of the Sp1 site and/or CCAAT box in vTR expression during tumorigenesis (Fig. 2C). However, further studies are needed in order to address the importance of these sites in vTR expression *in vivo*. These findings indicated the possible role of DNA methylation in the activity of the vTR promoter at the key steps of GaHV-2 infection and were further investigated.

### Impact of methylation on the activity of the vTR promoter.

**FIG 3:**
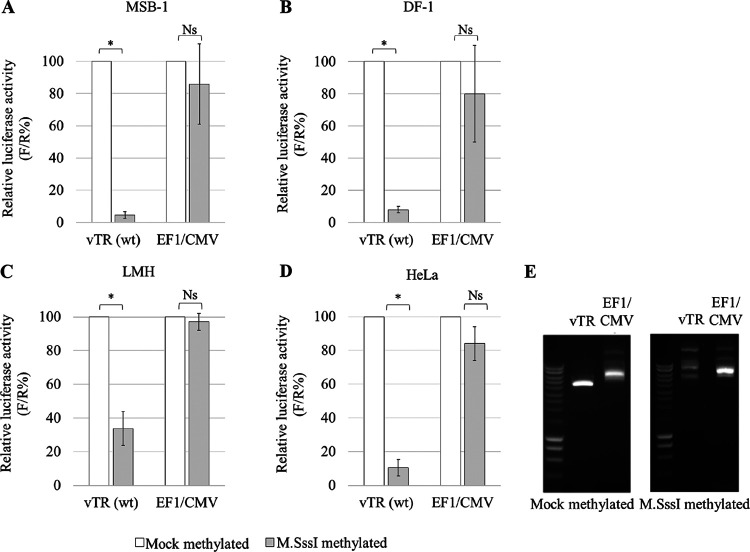
Effect of methylation on the activity of the vTR promoter. After hypermethylation with DNA methyltransferase (M.SssI), the relative activity of the vTR promoter was measured in the cell lines MSB-1 (A), DF-1 (B), LMH (C), and HeLa (D). Luciferase activity was quantified with the dual-luciferase reporter assay system, and the results are presented as firefly/Renilla percentage (F/R%). The hybrid promoter EF1/CMV, known to be insensitive to methylation, was used as a control. (E) The methylation-sensitive restriction enzyme HpaII was used to confirm the success of hypermethylation on vTR plasmid constructs. Effect of restriction digestion with HpaII is shown for mock-methylated plasmids and M.SssI-methylated plasmids. Significant differences on triplicates were assessed with Student's *t* test for the vTR promoter: ns, not significant; *, *P* ≤ 0.05.

To address the effect of methylation on the activity of the vTR promoter, a luciferase promoter-reporter assay was performed. A plasmid backbone was used that lacks CpG sites except for the ones present in the target promoters. After hypermethylation with the CpG methyltransferase M.SssI, the relative activity of the vTR promoter was measured in three chicken cell lines, MSB-1 (Fig. 3A), DF-1 (Fig. 3B), and LMH (Fig. 3C), as well as human HeLa cells (Fig. 3D). Hypermethylation of the vTR promoter led to a significant decrease in relative activity in all cell lines, compared to nonmethylated ones. In the case of the control promoter EF1/CMV, devoid of CpG dinucleotides and thus insensitive to methylation, no significant changes in activity between methylated and nonmethylated promoters were observed (Fig. 3). The efficiency of M.SssI treatment on the vTR promoter was confirmed by digestion with the methylation-sensitive restriction enzyme HpaII. Nonmethylated promoters were digested with HpaII, while no enzymatic digestion was observed in the presence of methylated promoters (Fig. 3E).

### Methylation process masks the effect of site-directed mutagenesis of the c-Myc binding sites.

**FIG 4:**
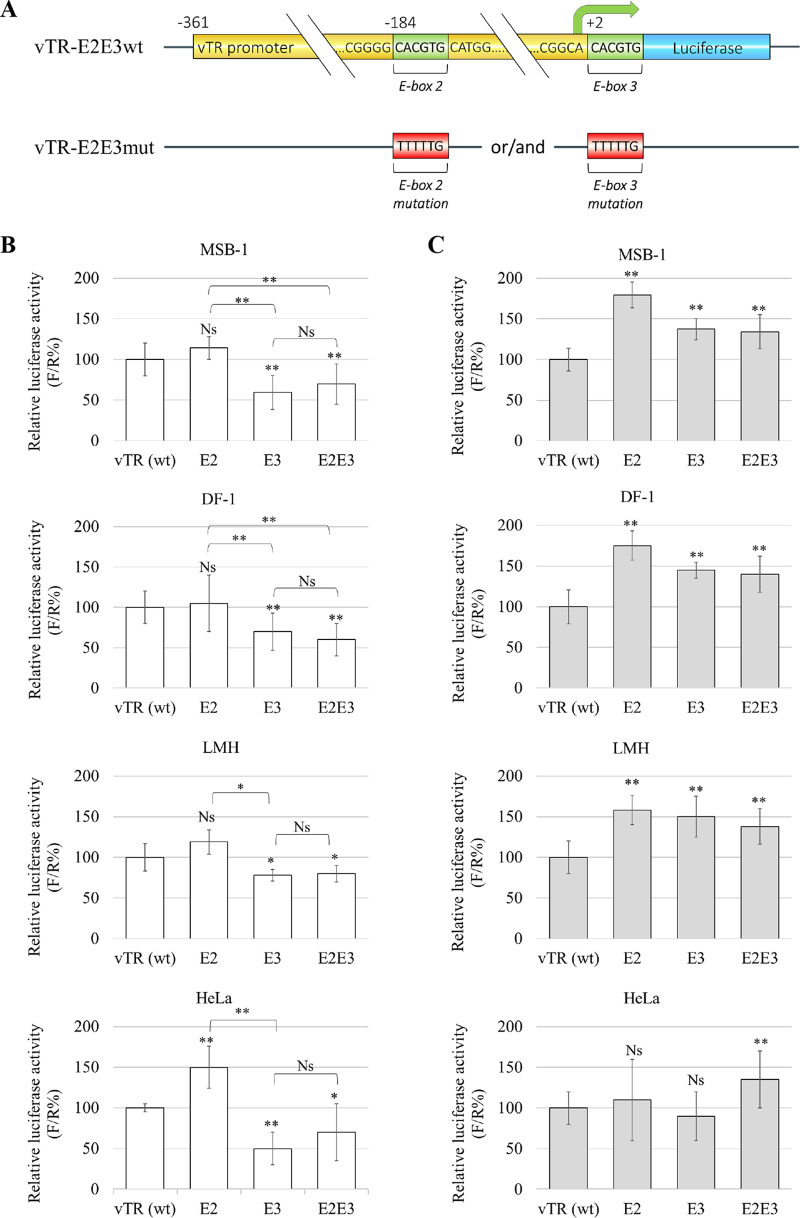
Effect of methylation on mutated vTR promoter constructs. (A) Specific response elements of the vTR promoter were studied through the mutagenesis of the E-box binding sites for the c-Myc transcription factor. E-box mutations were generated by PCR site-directed mutagenesis to obtain E2, E3, and E2E3 mutants. Luciferase activity was quantified with the dual-luciferase reporter assay system and the results are presented as firefly/Renilla percentage (F/R%). The relative activity of mutated vTR promoters was measured in MSB-1, DF-1, LMH and HeLa cell lines in nonmethylated conditions (B) and after hypermethylation with the DNA methyltransferase M.SssI (C). Significant differences on triplicates were assessed with Student's *t* test for the vTR promoter: ns, not significant; **, *P* ≤ 0.005.

Our previous study of CpG methylation mapping revealed specific methylation changes in functional c-Myc REs of the vTR promoter. To further characterize the effect of methylation on c-Myc transcription factor binding sites, functional c-Myc REs of the vTR promoter were mutated by site-directed mutagenesis, obtaining E-box 2 (E2), E-box 3 (E3), and double E2E3 mutants (Fig. 4A). The luciferase reporter promoter assay was used to study the activity of mutated versus wild-type vTR promoters (Fig. 4A). In chicken cell lines MSB-1, DF-1, and LMH, in unmethylated conditions, luciferase activity showed the E2 mutation alone did not affect vTR expression (Fig. 4B). On the other hand, E3 and E2E3 mutations showed repression of vTR promoter activity compared to the wild-type promoter (Fig. 4B). There were no significant differences between the relative luciferase activities measured for the E3 mutated vTR promoter and the double mutant E2E3. The significant difference between the relative luciferase activity of the mutated E2 promoter and that of the double mutant E2E3 suggests the E3 box is more functional in MSB-1, DF-1, and LMH cells (Fig. 4B). For the HeLa cell line, used as a nonhomologous GaHV-2 control, only the E2 mutation in the vTR promoter did not follow the same activity pattern, highlighting the difference between human and chicken cell lines and response elements (Fig. 4B).

More intriguingly, the luciferase activity measured for methylated and mutated E2, E3, and E2E3 promoters in MSB-1 and DF-1 cells, as well as in LMH cells, was significantly higher than that in the methylated wild-type promoter (Fig. 4C). In the HeLa cell line, there were no significant changes in the activity of the methylated promoters, except in the double E-box mutant (Fig. 4C). The efficiency of M.SssI treatment for mutated vTR promoters was confirmed by digestion using the methylation-sensitive restriction enzyme HpaII. Nonmethylated promoters were digested with the HpaII. However, in the presence of methylated promoters, lack of the enzymatic digestion was observed (data not shown). These results showed the E3 box is involved in regulating the activity of the vTR promoter and indicated that the methylation process masked the effect of site-directed mutagenesis of the c-Myc binding site.

### Generation and replication properties of the recombinant viruses.

**FIG 5:**
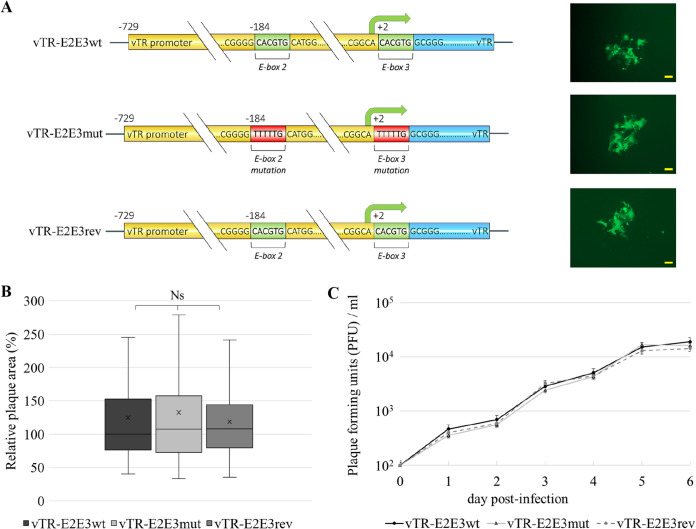
Replication properties of recombinant constructs showed no significant difference between wild- type, mutated, and revertant recombinant viruses. (A) Mutations in the E-boxes of the vTR promoter were introduced using two-step Red-mediated mutagenesis using a bacterial artificial chromosome (BAC) of the highly oncogenic RB-1B strain lacking part of the internal repeat long region (pRB-1BΔIRL). Using the mutated BAC as a backbone, revertant bacmid was produced containing the wild-type sequence. Next to each recombinant virus, a representative image of viral plaques is shown (scale bar 100 μm). (B) Relative plaque areas were calculated at 6 days postinfection using Image J software and are shown as box plots with minimums and maximums. Results are shown as the means of three independent experiments revealing no significant difference in viral replication properties between wild-type (vTR-E2E3wt), mutant (vTR-E2E3mut), or revertant (vTR-E2E3rev) viruses (assessed by ANOVA). (C) Multistep growth analysis assay confirmed the introduced E-box mutations did not affect replication of the constructs. Average titers of an independent experiment performed in triplicate are shown with standard deviations (*P* > 0.05, Kruskal–Wallis test).

To assess the role of the CpG sites within functional c-Myc REs in GaHV-2 replication and pathogenesis, we generated recombinant virus containing a mutation in the c-Myc REs (vTR-E2E3mut) by two-step Red-mediated mutagenesis using the highly oncogenic RB-1B strain lacking part of the internal repeat long region (pRB-1BΔIRL) as a backbone. In addition, the original vTR promoter sequence was restored by obtaining a revertant virus (vTR-E2E3rev) (Fig. 5A). Constructed recombinant viruses were verified by restriction fragment length polymorphism (RFLP) using BamHI and KpnI restriction enzymes, PCR, and sequencing of the vTR promoter locus. Additionally, to confirm the integrity of the recombinant viral genome after several mutagenesis steps, the final mutant and revertant bacmid clones were analyzed with high-throughput sequencing. The analysis revealed that mutagenesis of functional c-Myc response elements (E-box 2 and E-box 3) was successful, and confirmed the revertant construct had the same sequence as the wild-type reference.

### Mutation of CpG sites within c-Myc response elements does not affect GaHV-2 replication *in vitro*.

To assess if the mutation of functional c-Myc REs influenced virus replication, replication properties of recombinant and revertant viruses were assessed by plaque size assays (Fig. 5B) and multistep growth kinetics (Fig. 5C). Both assays indicated that replication of the mutant virus was comparable to wild type and revertant and not altered by the c-Myc REs mutations *in vitro*. Thus, only mutant (vTR-E2E3mut) and revertant (vTR-E2E3rev) recombinant viruses were used for animal experiments.

### Mutation of CpG sites within c-Myc response elements results in a phenotype of severely impaired tumor formation.

To investigate the involvement of the c-Myc oncoprotein and the importance of its binding on the E-box sites for tumor development, 2-day-old B^13^B^13^ chickens were infected intramuscularly with 2,000 PFU of either mutant (vTR-E2E3mut) or revertant (vTR-E2E3rev) inoculum. During infection, the onset of characteristic clinical symptoms was monitored.

**FIG 6:**
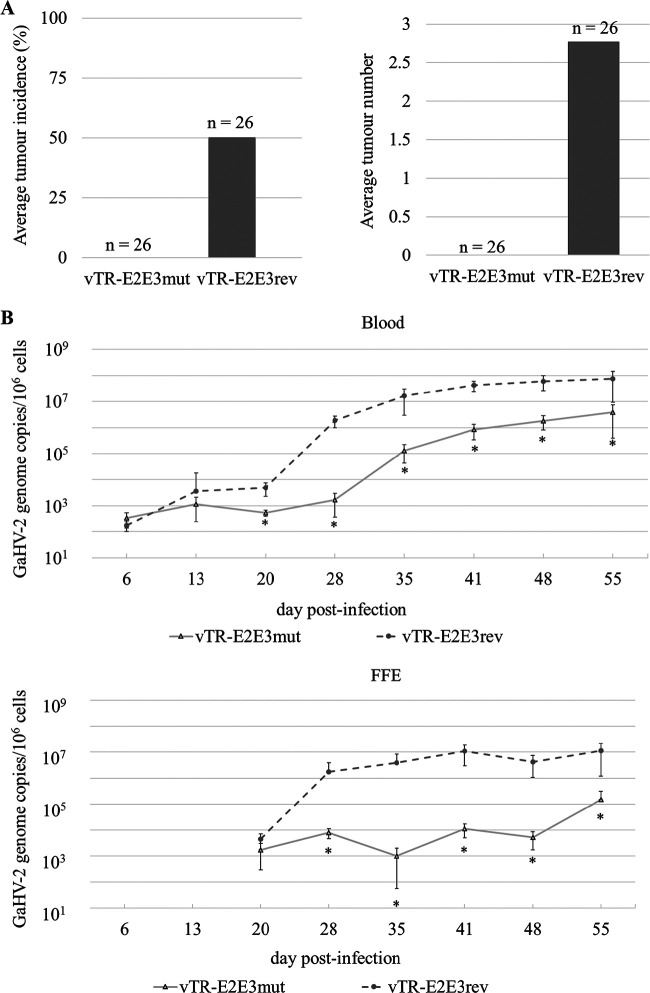
Mutation of the CpG sites within c-Myc response elements in the vTR promoter results in a specific disease phenotype I. (A) At 55 days postinfection, all animals were euthanized and autopsies were performed to determine tumor formation in internal organs. While 50% of animals in the revertant group (vTR-E2Erev) developed visible tumors, the animals challenged with the mutated recombinant virus (vTR-E2E3mut) had no tumors recorded. The average number of gross tumors per animal infected with recombinant viruses was calculated. An average of 2.77 tumors was recorded for the chickens challenged with the revertant virus (vTR-E2E3rev). (B) qPCR detecting GaHV-2 genome copies in the blood and feather follicle epithelium (FFE) of chickens infected with mutant (vTR-E2E3mut, *n* = 26) or revertant (vTR-E2E3rev, *n* = 26) viruses. The means of GaHV-2 genome copies per million cells are shown for the indicated time points. Total RNA was isolated from peripheral blood leukocytes (PBLs) at 55 dpi, and RT-qPCR was performed.

Furthermore, to assess the effect of introduced mutations on tumor propagation, the number of visceral organs with visible tumor lesions and the number of tumors were recorded at 55 dpi. Strikingly, for the vTR-E2E3mut virus, no animals developed visible tumors, while 50% of animals infected with the revertant virus did (Fig. 6A). The average of 2.77 tumors per animal was recorded for the group infected with the revertant virus (Fig. 6A). The total number of tumors per screened internal organs indicated the majority of tumors developed in the kidneys and the livers of the vTR-E2E3rev-infected animals (data not shown). In addition, the accumulation of adipose tissue was visible around the heart and liver of animals that had developed visible gross tumors.

### Mutation of CpG sites within c-Myc response elements results in lower viral loads in infected animals.

To determine if the recombinant viruses efficiently replicated in infected chickens, viral genome copies were quantified from the whole blood and feather follicle epithelium (FFE) at specific time points. Monitoring the viral load evolution throughout GaHV-2 infection demonstrated that the introduced mutations in functional c-Myc REs affected total viral loads levels, which were significantly lower compared to the revertant virus (Fig. 6B). Furthermore, the quantification of viral copy numbers in FFE, starting at 20 dpi, also showed decreased viral loads compared to the revertant virus, indicating a reduction in mature virion release in the FFE (Fig. 6B).

### Mutation of CpG sites within c-Myc response elements affects relative vTR expression and results in decreased telomerase activity.

**FIG 7:**
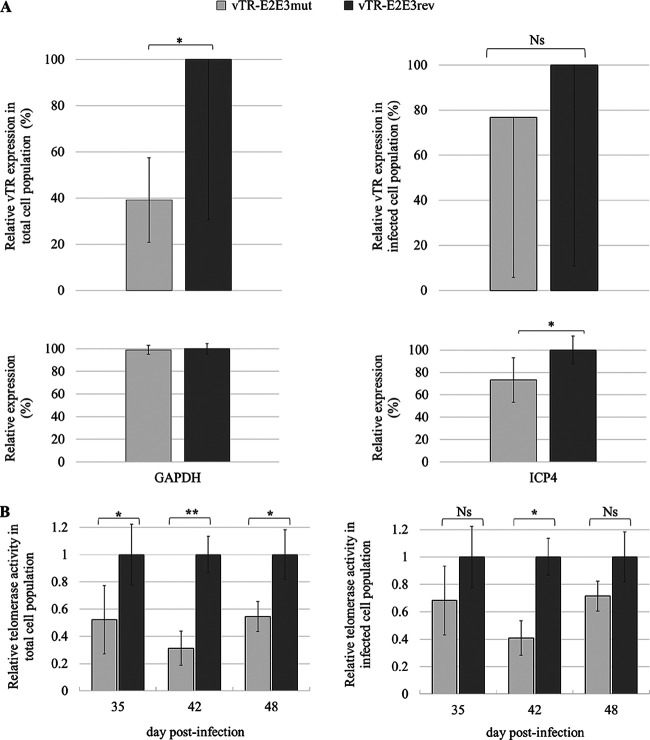
Mutation of the CpG sites within c-Myc response elements in the vTR promoter results in a decrease in relative vTR expression and relative telomerase activity. (A) Relative vTR expression was obtained using the Livak method and is shown relative to the cellular GAPDH control gene (*P* ≤ 0.05, Student’s *t* test). Relative expression of control gene GAPDH in the peripheral blood leukocytes (PBLs) infected with the different viruses was not statistically different (*P* > 0.05, Student’s *t* test). In addition, vTR expression is shown relative to the viral ICP4 control gene (*P* ≥ 0.05, Student's *t* test), yielding a similar tendency in vTR expression reduction as shown for results obtained with GAPDH as a control. Relative expression of the control ICP4 gene in PBLs infected with the different viruses was statistically different (*P* < 0.05, Student’s *t* test). The means of three independent experiments with standard deviations are shown. (B) PBLs were extracted from the blood of eight random animals infected either with mutated or revertant recombinant viruses. Extracted PBLs from each group were pooled and total protein extract was submitted to semiquantitative fluorescence-based telomeric repeat amplification protocol assay. The relative telomerase activity was normalized to the gene coding for the major viral oncoprotein Meq. Relative telomerase activity significantly decreased at day 42 postinfection in the animals infected with the virus bearing mutations in the functional c-Myc response elements of the vTR promoter and demonstrated similar tendencies at days 35 and 48 postinfection. Student’s *t* test: ns, not significant; *, *P* ≤ 0.05; **, *P* ≤ 0.005.

To investigate the effect of the inserted mutation on vTR expression, we performed reverse transcriptase quantitative PCR (RT-qPCR) assays at 55 dpi on total RNA extracted from PBLs of the animals infected with either vTR-E2E3mut or vTR-E2E3rev recombinant virus. Interestingly, relative vTR expression was reduced by 2.5-fold in the animals infected with the vTR-E2E3mut virus compared to those infected with vTR-E2E3rev (Fig. 7A). GAPDH was used to normalize the data, and the expression of GAPDH was comparable between the two groups (Fig. 7A). In addition, relative vTR expression was normalized relative to the viral ICP4 control gene, resulting in the loss of the significant reduction in vTR expression between the conditions. However, the tendency of vTR expression reduction was preserved, as shown for results obtained with GAPDH as a control (Fig. 7A).

In order to assess the effect of the c-Myc binding site mutations on vTR involvement in the regulation of telomerase activity, peripheral blood leukocytes (PBLs) were extracted from B^13^B^13^ chickens infected with either the revertant or mutant recombinant virus at 35, 42, and 48 dpi. Full protein extract of 1 × 10^6^ PBLs was analyzed using the semiquantitative fluorescence-based telomeric repeat amplification protocol (TRAP) assay (Fig. 7B). Introduced c-Myc REs mutations in the vTR promoter resulted in 1.9, 3.2, and 1.8-fold decreases in telomerase activity, respectively, compared to the internal amplification standard. Additionally, the relative telomerase activity was normalized to the gene coding for the major viral oncoprotein Meq that allowed a more precise readout due to the differences in the number of viral genomes observed between the animals infected with mutant or revertant viruses. The normalization with the viral gene resulted in 1.5, 2.4, and 1.4-fold decreases in relative telomerase activity measured at 32, 42, and 48 dpi, respectively (Fig. 7B). The relative telomerase activity in noninfected control animals, measured from 9 to 30 days posthatching, was assessed for a basal activity reference. The highest basal telomerase activity was recorded at day 9 compared to the activity measured at 16, 23, and 30 days posthatching. Starting from day 16, the telomerase activity measured in noninfected chickens stabilized and did not significantly change and, moreover, was significantly reduced compared to the telomerase activity measured in infected chickens (data not shown).

## DISCUSSION

Gallid herpesvirus type 2 (GaHV-2) is a highly oncogenic alphaherpesvirus that infects chickens, causing paralysis, immunosuppression, and fatal lymphoma in susceptible animals (17). GaHV-2 establishes latent infection in CD4^+^ T lymphocytes, in which it integrates the viral genome into the telomeres of host chromosomes (12, 18, 19). The integrated virus genome is maintained in the telomeres and mobilized during reactivation. Aside from latency, telomere integration also plays an essential role in tumor formation (20).

One of the main characteristics of herpesviruses is their ability to establish a latent infection during which most of the viral genes are silenced, resulting in no viral progeny production and viable host cells. However, the virus can reactivate and produce new virions if conditions in the cell change (21). A crucial factor in the regulation of gene expression associated with different phases of the viral cycle in herpesviruses is epigenetic modifications (18, 21). In this context, we have studied the effect of the epigenetic changes on the promoter of the vTR gene encoding the telomerase RNA subunit. We performed methylation mapping on the vTR promoter and demonstrated a reduction of methylation signatures after viral reactivation *in vitro*. These results correspond to previous data published on GaHV-2 methylation changes (22), as well as in other herpesviruses (21, 23, 24).

In this study, we observed an increase in telomerase activity after viral reactivation *in vitro*. This resulted in DNA demethylation of the GaHV-2 genome and indicated that DNA methylation status is associated with telomeric transcription in a GaHV-2-transformed cell line. This result is in agreement with previous studies observing high telomerase activity in tumor cell lines in comparison to normal lymphocyte cells (25). Telomere maintenance is necessary for unlimited cancer cell proliferation and, moreover, previous studies suggested that telomerase could promote tumorigenesis independently of telomere elongation (26, 27). Increased telomerase activity has been detected in cells infected with a variety of herpesviruses (10, 27–29); however, none of these viruses harbors any of the telomerase components except GaHV-2. Cancer-associated human herpesviruses have also been found to upregulate telomerase activity upon cell infection. In Epstein-Barr virus (EBV)-immortalized B lymphocytes, telomerase activity was variable (30). Similar results were observed in a nasopharyngeal carcinoma cell line expressing the EBV-encoded latent membrane protein 1 (LMP1). LMP1 expression was correlated with increased hTERT promoter activity and protein levels, suggesting enhanced hTERT transcription as a mechanism for telomerase upregulation (31). A similar effect was identified in cells expressing the latency-associated nuclear antigen (LANA) gene of Kaposi’s sarcoma herpesvirus (KSHV/HHV-8) (32, 33). These data implicate telomerase activation as a common mechanism for herpesvirus tumorigenesis and increases in telomerase activity during herpesvirus infections (34).

*In vitro* replication properties of recombinant viruses supported previous studies showing that vTR is dispensable for lytic GaHV-2 replication (10, 35). Furthermore, we assessed the impact of c-Myc RE mutations on virus-induced tumor development. In addition, numerous studies have indicated that wild-type and revertant viruses replicate in similar ways *in vivo* and result in similar output in tumorigenesis (10, 12, 35), strengthening our approach to use only revertant virus as a control. Strikingly, animals infected with the mutant virus showed abrogation of tumorigenesis, while half of the animals infected with revertant virus developed gross tumors in visceral organs. It was previously demonstrated that vTR contributes to GaHV-2-induced lymphomagenesis, where complete deletion of vTR resulted in significantly reduced tumor incidence without affecting virus replication *in vivo* (10). However, no previous studies reported the differences in the distribution of the tumors in tissues. Surprisingly, viral loads in susceptible B^13^B^13^ chickens infected with recombinant virus bearing c-Myc RE mutations was significantly lower compared to the revertant virus, starting from 20 dpi. This observation could be explained by the fact that during the productive phase (~4 to 12 dpi), the viral titers are usually very low; however, that changes at later time points where most if not all viral genome copies are detected from transformed/tumor cells. Thus, the viral loads we detected were from the total number of GaHV-2-transformed T lymphocytes, the result that is supported by the lack of visible tumors in the same group. A similar tendency, though not significant, was previously observed by Trapp et al. (10). In addition, the significantly lower levels of viral load observed in the feather follicle epithelium (FFE) backs up previous findings, confirming that a lower number of transformed T lymphocytes circulating in the blood establishes weaker secondary productive infection and viral shedding from the FFE. Moreover, even though complete deletion of vTR severely impaired tumor formation, no total abrogation was previously demonstrated (10, 35). However, the total lack of visible tumors we observed might be due to differences in the major histocompatibility complex-B (MHC-B) between this line and the susceptible chicken lines used previously, along with the duration of the experiment. As demonstrated by Kheimar et al. (35), the first tumor incidence in the animals infected with a virus lacking both vTR copies was recorded around 56 dpi. Only 30% of infected animals developed tumors until 91 dpi, while wild-type RB-1B caused tumors in around 90% of animals. Moreover, the study using the same B^13^B^13^ chicken line demonstrated that 83% of disease incidence was reached after 90 days of infection (36). Thus, there is a high probability that animals infected with the recombinant virus bearing c-Myc RE mutations in the vTR promoter would eventually develop visible tumors over time. Furthermore, it was also demonstrated that only 36% of birds infected with the virus containing a complete deletion of vTR developed lymphomas, compared with 88% of birds infected with the RB-1B strain. In addition, lymphomas induced by viruses harboring at least one intact copy of vTR predominantly disseminated to multiple organ sites, whereas the majority of lesions induced by virus lacking both vTR copies affected no more than two organs (10). Furthermore, our findings indicated that mutations of c-Myc binding sites in the vTR promoter have a significant repressive effect on vTR expression during GaHV-2 infection compared to the revertant virus normalized with the chicken control gene, as well as the tendency toward inhibition of vTR expression when normalized to a viral gene, indicating that functional c-Myc REs are involved in the regulation of vTR expression. These results support our previous observation of 50% of animals developing gross tumors in the revertant group. In addition, this observation is consistent with previous reports demonstrating that vTR expression is not only crucial for GaHV-2 lymphomagenesis, but expression levels are necessary for GaHV-2 tumorigenic function. It was shown that vTR expression through its promoter is essential for GaHV-2 lymphomagenesis, revealing that tumor formation induced by recombinant viruses expressing vTR at lower levels was significantly inhibited (13). Furthermore, it was indicated that overexpression of chicken cellular TR (chTR) promoted tumor formation as efficiently as vTR, indicating that expression levels of chTR/vTR are of critical importance for tumor development (35).

We demonstrated that the relative telomerase activity measured in total peripheral blood leukocytes is significantly lower in the animals infected with the virus containing mutations in the c-Myc REs of the vTR promoter compared to the revertant virus, consistent with the significantly lower vTR expression. Moreover, animals infected with both GaHV-2 recombinant viruses demonstrated significantly higher relative telomerase activity than in noninfected chickens. Our findings correlate with previously published data that showed vTR enhances telomerase activity compared to the cellular TR (9, 14). In addition, it was previously demonstrated that vTR-mediated telomerase activity contributes to the rapid onset of disease, but not tumor formation (11). Therefore, the observed lack of Marek’s disease incidence in the animals infected with the vTR-E2E3mut virus is likely due to the observed lower relative telomerase activity. However, the relative telomerase activity measured in the infected cell population resulted in the loss of significant change at days 35 and 48 postinfection, preserving the similar tendencies observed in the total cell population. These results indicate that the observed reduction in telomerase activity could be due to the different viral loads and thus viral activity during the infection induced with the mutant or revertant virus or, alternatively, the consequence of reduced vTR expression and thus lower interaction with TERT.

Furthermore, the extreme phenotype observed in our study could indicate other possible regulatory events involved. Since this implication was not studied before in the same context, further studies are needed to obtain a full mechanistic picture of vTR promoter regulation. In addition, it is essential to mention that other vTR functions independent of the telomerase complex could play a role in tumorigenesis. It was shown that vTR, similarly to EBV EBER-1 (37) and human TR (38), interacts with the cellular ribosomal protein L22 (RpL22) (11). RpL22 plays a vital role in T-cell development and lymphoma formation (39, 40), the main targets of GaHV-2 transformation. The interaction of EBER-1 with RpL22 results in relocalization of RpL22 from the nucleolus to the nucleoplasm and is associated with enhanced potential for cellular proliferation (41). The described vTR/RpL22 interaction indicates an alternative GaHV-2 transformation mechanism that may be similar to that demonstrated for EBER-1 (11). However, further studies are needed in order to understand how these interactions contribute to tumorigenesis in general.

We showed that DNA methylation could play a role in the restriction of specific genes, such as vTR, during the GaHV-2 replication/latent cycle. Specific changes in the methylation patterns were observed throughout the vTR promoter region *in vitro*, especially in the areas surrounding functional c-Myc REs (E-box 2 and E-box 3), which serve as binding sites for proteins of the Myc/Mad/Max transcription factor family and act as crucial positive regulators of cell proliferation and death (42, 43). Previous studies have shown that c-Myc can induce telomerase activity through the transcriptional activation of hTERT (44, 45). In 2007, Shkreli et al. (14) showed that c-Myc activates transcription of the vTR gene and binds to the vTR promoter sequence in a GaHV-2-transformed cell line. The interaction of c-Myc with the vTR promoter E-boxes is involved in the higher levels of vTR expression observed during GaHV-2-induced lymphomagenesis, and EBS and E-box 2 act together with E-box 3 to regulate vTR expression in an MSB-1 cell line. Moreover, the results for DNA methylation patterns *in vivo* were obtained from a total population of isolated peripheral blood leukocytes and showed random changes in methylation patterns, even as we observed tendencies in methylation pattern changes in and surrounding functional c-Myc response elements. This result indicated that cell sorting could overcome these misleading observations, highlighting the limits of our approach.

Furthermore, we demonstrated that DNA hypermethylation actively represses the transcriptional activity of the vTR promoter in the recombinant plasmids. DNA methylation is one of the epigenetic marks associated with repression of gene expression. It has been suggested that during herpesvirus infection, the viral genome is subjected to a biphasic methylation cycle. Widely methylated during the viral latency, it returns to an unmethylated state during lytic viral replication (15, 46, 47). The data obtained here corroborate previous studies showing that DNA methylation represses the specific transcription of promoters during the latent phase (48). The same effect has also been observed for EBV (49). However, in some cases, it has been shown that DNA methylation could have an opposite effect and thus be associated with transcriptional activation. For example, the reactivation of the EBV virus via the overexpression of the ZTA viral protein, which binds preferentially to methylated sites (50). As mentioned before, the c-Myc transcription factor plays a role in the expression of the vTR gene during the latency phase of GaHV-2. The vTR promoter has three c-Myc-binding sites, namely E-box 1, E-box 2, and E-box 3, and it has been confirmed that E-box 1 is not functional (14). For this reason, the study of the effect of methylation on c-Myc transcription factor binding sites was conducted on E-boxes 2 and 3. The results showed that the mutation of E-box 2 does not affect the transcriptional activity of the unmethylated vTR promoter, contrasting with mutations in E-box 3 and the double E2E3 mutation that are associated with a decrease in the expression of the vTR promoter. Similar results were obtained for LMH cells (14). These results confirm that E-box 3 is a *cis*-regulatory element involved in vTR transcription. However, the double E2E3 mutation did not induce complete inhibition of transcription, which suggests the involvement of other transcription factors regulating activity of the vTR promoter. Indeed, the bioinformatic analysis of the vTR promoter showed a multitude of binding sites for different transcription factors, among which the binding site for the Ets transcription factor is also involved in the regulation of vTR transcription in the GaHV-2 transformed MSB-1 cell line (14).

For the methylated promoters, the mutation of E-boxes had an opposite effect. The activity of the mutated and methylated vTR promoter was significantly higher than that of the methylated wild-type promoter. These surprising results suggest that, contrary to observations made in an unmethylated situation, methylation appears to mask the effect of the mutation. The high activity of the methylated mutated promoters might be because, in the absence of E-box mutation, c-Myc would be the main transcription factor for the activation of the vTR transcription. The mutation of the c-Myc binding sites could induce the recruitment of other transcription factors that are insensitive to the methylation. Indeed, a series of factors have been described as being insensitive to methylation, e.g., Sp1, FCT, YY1, and C/EBP alpha-factor (50–52). According to the results obtained in our study, it was expected that the activity of the mutated vTR promoter would be negatively influenced by methylation. That we obtained the opposite effect might be explained by the fact that during GaHV-2 infection, the regulation of the vTR promoter is not only dictated by the c-Myc but the result of the association of several factors, among which the Meq viral protein could have an important role. Indeed, the Meq protein has a central role in the regulation of the expression of many genes during the different phases of the GaHV-2 life cycle and could positively regulate the expression of vTR. The Meq protein, via the Pro-Leu-Asp-Leu-Ser motif, could bind the C-terminal-binding protein (CtBP), which is a known corepressor involved in the regulation of cell proliferation cell growth and apoptosis. The binding of Meq to CtBP inhibits CtBP/E2F protein interactions that could result in E2F binding to its consensus sequence present on the vTR promoter and induce vTR transcription (2, 53). The viral oncoprotein Meq also has the leucine zipper binding site (B-ZIP) for the c-Jun protein. The Meq/Jun heterodimer could induce transcription of vTR by binding to the AP-1 site present at the vTR gene promoter (54).

Furthermore, the noticeable differences of this approach compared to *in vivo* observations regarding the effects of mutations in c-Myc response elements must be addressed. The effect of mutations introduced in the vTR promoter was established in an isolated context that was highly controlled in the cell lines. On the other hand, the *in vivo* conditions, due to their complex interaction with viral and cellular machinery present a unique environment that is less comparable to the *in vitro* situation, making it difficult to predict the results of introducing the highly controlled *in vitro* assay into the animal model.

In conclusion, our study provides further characterization of the c-Myc response elements within the vTR promoter and their importance in the regulation of vTR expression and, moreover, vTR involvement in GaHV-2-induced tumorigenesis.

## MATERIALS AND METHODS

### Cell lines.

The chicken embryo fibroblasts (CEFs) infected with the RB-1B strain were used for the productive phase of the viral life cycle. The CEFs were obtained from 12-day-old chicken embryos treated by trypsinization (Lonza). The primary CEFs were cultured in Dulbecco’s modified Eagle medium (DMEM) (Lonza) supplemented with 2.5% fetal bovine serum, 1.25% chicken serum, 1% penicillin (50 units/ml) and streptomycin (50 μg/ml), 1% fungizone (GIBCO), and 1.475 g/liter tryptose phosphate (Sigma). Four days after primary CEF cultures, cells were passaged to produce secondary CEFs. Secondary CEFs were transfected with an infectious clone of the RB-1B bacmid using Lipofectamine 2000 (Invitrogen) according to the manufacturer’s protocol.

Embryonic stem cell-derived line-1 (ESCDL-1), a mesenchymal cell line used as a GaHV-2 productive infection model (55), was cultured in a supplemented Dulbecco’s modified Eagle medium (DMEM F12 1:1) supplemented with 10% fetal bovine serum, 1% penicillin (50 units/ml) and streptomycin (50 μg/ml), 1% nonessential amino acids, and 1% sodium pyruvate. Cells were maintained at 37°C under 5% CO_2_. This cell line was kindly provided by Caroline Denesvre (INRA, Tours, France).

The latently infected and transformed MSB-1 cell line, derived from a GaHV-2 induced lymphoma (56), was cultured in RPMI 1640 medium (Gibco). Reactivation of the virus in the MSB-1 cells was induced by the treatment with 5 μM 5-azacytidine (Merck), an inhibitor of the DNA methyltransferase, described as a reactivation stimulus for GaHV-2 (57).

The avian fibroblast cell line DF-1, derived from primary chicken embryonic fibroblasts, was cultured in DMEM medium (Lonza). The LMH cell line established from chicken liver carcinoma epithelial cells (58) was cultured with 0.2% gelatin to maximize cell adhesion in DMEM medium (Lonza).

The human epithelial HeLa cell line, derived from a cervical carcinoma and transformed by human papillomavirus type 18 (59), was maintained in EMEM medium (Lonza). All media were supplemented with 10% fetal bovine serum, 5% chicken serum (except EMEM), 1% of nonessential amino acids, and 1% penicillin (50 units/ml) and streptomycin (50 μg/ml).

The MSB-1, DF-1, and LMH cell cultures were maintained at 41°C while HeLa cells were kept at 37°C, all under 5% CO_2_.

### Telomeric repeat amplification protocol assay.

Telomerase activity of 1 μg of protein extracted from the MSB-1 cells or peripheral blood leukocytes (PBLs) extracted from the blood of infected chickens was quantified using the semiquantitative fluorescence-based telomeric repeat amplification protocol (TRAP) assay, as previously described (9). The PCR was carried out using tetramethylrhodamine (TAMRA)-labeled forward TS and CX-ext as reverse primers as initially described (Table 1) (60). An internal amplification standard (ITAS) was included to verify PCR amplification efficiency. ITAS was prepared by PCR as previously described (61) using primers presented in Table 1. The telomerase amplification PCR and the relative telomerase activity were calculated as previously described (25). Additionally, the relative telomerase amplification measured *in vivo* was normalized using the relative expression of a major viral oncoprotein, Meq, in order to have a more precise way of interpreting the data in the infected cell population.

**TABLE 1:** Primers used for the telomerase activity, DNA methylation patterns, and sequencing

Target/Construct	Orientation	Sequence (5′–3′)
Primers used for TRAP assay^*a*^		
ITAS	Forward	AATCCGTCGAGCAGAGTTGTGAATGAGGCCTTC
	Reverse	CCCTTACCCTTACCCTTACCCTAATAGGCGCTCAATGTA
Telomeric amplification	Forward	**(TAMRA)**-AATCCGTCGAGCAGAGTT
	Reverse	GTGCCCTTACCCTTACCCTTACCCTAA
Primers used for RT-qPCR confirming viral reactivation		
GaHV-2-VP5	Forward	CAAGGGGATCCCGCATATCCATTTCG
	Reverse	CAGGGGTCCTCGGTCAATTCGGTGG
U6	Forward	CTCGCTTCGGCAGCACATATAC
	Reverse	TTTGCGTGTCATCCTTGCGC
Primers used for nested PCR for BGSA		
vTR promoter	Forward-1	TTAATATTTTYGATTAGGGTTAG (bisulfite modifed); TCAATACCTCCGATTAGGGTTA (original)
	Reverse-1	AACAAACAATTATACACCTACCT (bisulfite modifed); GACAGACAGTTGTACACCTGCCT (original)
	Forward-2	GATTAGGGTTAGATATAGYGGAG (bisulfite modifed); GATTAGGGTTAGACACAGCGGAG (original)
	Reverse-2	CACCTACCTACACTACTACATCC (bisulfite modifed); CACCTGCCTGCACTACTACATCC (original)
Primers used for c-Myc response elements PCR directed mutagenesis^*b*^		
E-box 2 mutation	Forward-1	**(5′PstI)** **GTGCAG**CCCTAACCCTAACCCCCCAAATTTCACC
	Reverse-1	ACGCCCCATGTTTTTGCCCCGCCCCTTCCTG
	Forward-2	GGGCGGCAAAAACATGGGGCGTGGCGGGA
	Reverse-2	**(5′HindIII)** **AAGCTT**GCCTTCCACCCGCCACGTGTG
E-box 3 mutation	Forward	**(5′PstI)** **GTGCAG**CCCTAACCCTAACCCCCCAAATTTCACC
	Reverse	**(5′HindIII)** **AAGCTT**GCTTCCACCCGCCAAAAATGCCGGGGGAACC
Primers used for screen PCR and Sanger sequencing		
pGEMT-easy vector insert	Forward-M13	TGTAAAACGACGGCCATG
	Reverse-M13	CAGGAAACAGCTATGAC
pCpGL-Basic vector insert	Forward	GTTTATGTGAGCAAACAGCAG
	Reverse	GCATAGGTGATGTCCACCTC
vTR promoter	Forward	GTACACCTGCCTGCACTACT
	Reverse	GCGAGGACCCCAGGG

a TRAP, telomeric repeat amplification protocol; TAMRA, tetramethylrhodamine, a fluorescent label.

b Restriction sites are in bold, mutations are underlined.

Viral reactivation was monitored and confirmed by RT-qPCR measurement of relative expression of the major viral capsid protein VP5 at 48 h after the treatment with specified inhibitor. Relative expression levels were normalized against chicken small nuclear RNA U6 gene, using primers presented in Table 1.

### Bisulfite genomic sequencing assay and PCR.

The bisulfite genomic sequencing assay (BGSA) was used for 5-methylcytosine mapping, as described before (62). Bisulfite treatment was performed with the EZ DNA Methylation-Gold kit (Zymo Research) according to the manufacturer’s recommendations. Nested PCR was performed using the primers presented in Table 1, with the Epimark HotStart *Taq* DNA polymerase (New England BioLabs [NEB]). Produced amplicons were cloned into the pGEM-T Easy Vector system (Promega) followed by DNA sequencing (Table 1) and Geneious software analysis.

### Cell isolation from infected animals, magnetic cell sorting, and DNA extraction.

White Leghorn specific pathogen-free B^13^B^13^ chickens highly susceptible to GaHV-2 were used for animal experiments. The animals were housed in isolated biosecurity level 3 facilities at the Avian Virology and Immunology Service of Sciensano (Brussels, Belgium). Animals were injected intramuscularly at the age of 3.5 weeks with 1,000 PFU of the highly oncogenic GaHV-2 RB-1B strain, using an infected peripheral blood leukocyte (PBL) suspension. Blood samples were collected once a week and tumors were collected from euthanized chickens at 28 days postinfection (dpi). PBLs from anticoagulated blood and tumor tissue were isolated using a Histopaque-1077 density gradient (Sigma-Aldrich) according to the manufacturer’s recommendations at 14 dpi (representing the start of the latency) and 28 dpi (representing viral reactivation).

### PCR site-directed mutagenesis.

Site-directed mutagenesis was used to accurately induce specific mutations in the sequence of the vTR promoter. Mutations of the vTR promoter at the c-Myc transcription factor binding sites were performed by overlapping PCR using primer pairs described in Table 1. The backbone DNA used to generate the E2 and E3 mutations was the recombinant pGEM-T Easy vector (Promega) containing the wild-type vTR promoter. E-box 2 (E2) and E-box 3 (E3) mutations were introduced in two stages using primer pairs with overlapping fragments containing the mutation. Two overlapping fragments containing the mutation were initially constructed by PCR using two pairs of specific primers (Table 1). Using the same principle, the double E-box 2 and E-box 3 mutation (E2E3) was generated by introducing the E3 mutation on the vector bearing the E2 mutation that served as a PCR backbone, followed by confirmation by Sanger sequencing (Table 1).

### Plasmid construction and hypermethylation of plasmid DNA.

The pCpGL-Basic reporter vector, free from CpG dinucleotides and thus not sensitive to methylation, was used to study the activity of the vTR promoter. Wild-type and mutated vTR promoter constructs were cloned at the multiple cloning site upstream of the reporter gene coding for firefly luciferase. Plasmid pCpGL-EF1/CMV was used as the control vector. pCpGL-Basic and pCpFL-EF1/CMV vectors were generously provided by Michael Rehli (Department of Haematology and Oncology, University of Regensburg, Germany). The plasmid pRL-TK (Promega) containing the gene encoding Renilla luciferase was used to standardize the activity of firefly luciferase. The hypermethylation of the CpG dinucleotides of the different construct promoters was carried out using an M.SssI methyltransferase (NEB) and S-adenosyl-l-methionine (NEB) as described by the manufacturer. The plasmids were purified using phenol-chloroform-isoamyl alcohol (50:49:1) and confirmed by sequencing (Table 1).

### Cell transfection and dual-luciferase reporter assay.

Twenty-four hours before transfection, 1.5 × 10^4^ DF-1 and 3 × 10^4^ LMH and HeLa cells were seeded per well in 96-well plates. These cell lines were cotransfected with 150 ng of luciferase reporter constructs containing vTR wild-type or mutant E-box target sites and 30 ng of luciferase control vector using Lipofectamine 2000 (Invitrogen) according to the manufacturer’s protocols.

A suspension of 1 × 10^6^ MSB-1 cells in Nucleofector solution T (Amaxa Biosystems) was cotransfected with 1 μg of luciferase reporter constructs containing vTR wild-type or mutant E-box target sites and 40 ng of luciferase control vector. Cotransfection with electroporation was done using Nucleofector program X-001 (Nucleofector II, Amaxa) following the manufacturer’s instructions.

Luciferase activity was quantified in the Dual-Luciferase Reporter Assay system (Promega), according to the manufacturer’s protocol. Firefly and Renilla luciferase activities were measured 24 h after transfection. The firefly luciferase activity obtained for each sample was normalized by the corresponding Renilla luciferase activity. For the standardization of luciferase activity, the control vector pCpGL-EF1/CMV, from which the firefly luciferase gene is expressed under the control of the CMV promoter, was used.

Three independent experiments were carried out in triplicates. The significant differences between the analyzed promoter constructs were determined using the Student’s *t* test, and comparisons with *P* ≤ 0.05 were considered statistically significant.

### Construction of GaHV-2 recombinant viruses using two-step Red-mediated mutagenesis.

The recombinant viruses used in this study were generated using a two-step Red-mediated mutagenesis, as described previously (63). The backbone for recombinant GaHV-2 virus, carrying E-box 2 and E-box 3 mutation (vTR-E2E3mut), was the bacterial artificial chromosome (BAC) of the highly oncogenic RB-1B strain lacking part of the internal repeat long region that is rapidly restored upon virus reconstitution (pRB-1BΔIRL) (16). Thus, only one vTR copy had to be mutated, resulting in recombinant viruses harboring the desired mutation in both vTR loci upon reconstitution, as previously described (63). First, the E-box-2 mutation was inserted followed by E-box 3 mutation, as described previously, using primers shown in Table 2. Finally, a revertant virus (vTR-E2E3rev) was generated, restoring the original promoter sequence in the virus. All BAC constructs were screened by restriction fragment length polymorphism (RFLP) using multiple restriction enzymes, screening PCR, and sequencing of the vTR promoter locus (Table 1), as well as bacmid high-throughput sequencing, and were used to assess viral replication properties *in vitro* and were fully reconstituted afterward for animal studies.

**TABLE 2:** Primers used for the Red-mediated c-Myc response elements mutagenesis and c-Myc functional evaluation

Target/Construct	Orientation	Sequence (5′–3′)
Primers used for the construction of recombinant viruses^*a*^		
vTR-E-box_2-mutant	Forward	GATCCGATCCCGCAGACCCCGGCCCACAGGAAGGGGCGGGG**TTTTT**GCATGGGGCGTGGTAGGGATAACAGGGTAATCGATTT
	Reverse	GGAACTCCGCGGTCATTCATCTCCCGCCACGCCCCATGC**AAAAA**CCCCGCCCCTTCCGCCAGTGTTACAACCAATTAACC
vTR-E-box_3-mutant	Forward	GGAGGAAGCTACAAGAGCCCCACGCGGGGTTCCCCCGGCA**TTTTT**GGCGGGTGGAAGTAGGGATAACAGGGTAATCGATTT
	Reverse	CCTCCGATTAGGGTTAGACACAGCGGAGCCTTCCACCCGCC**AAAAA**TGCCGGGGGAACCGCCAGTGTTACAACCAATTAACC
vTR-E-box_2-revertant	Forward	CCGGATCCGATCCCGCAGACCCCGGCCCACAGGAAGGGGCGGGG**CACGT**GCATGGGGCGTGGTAGGGATAACAGGGTAATCGATTT
	Reverse	GAGTTTGGAACTCCGCGGTCATTCATCTCCCGCCACGCCCCATGC**ACGTG**CCCCGCCCCTTCCGCCAGTGTTACAACCAATTAACC
vTR-E-box_3-revertant	Forward	GGAGGAAGCTACAAGAGCCCCACGCGGGGTTCCCCCGGCA**CACGT**GGCGGGTGGAAGTAGGGATAACAGGGTAATCGATTT
	Reverse	CCTCCGATTAGGGTTAGACACAGCGGAGCCTTCCACCCGCC**ACGTG**TGCCGGGGGAACCGCCAGTGTTACAACCAATTAACC
Primers used for qPCR		
GaHV-2-VP5	Forward	CGTGTTTTCCGGCATGTG
	Reverse	TCCCATACCAATCCTCATCCA
	Probe	CCCCCACCAGGTGCAGGCA^*b*^
iNOS	Forward	GAGTGGTTTAAGGAGTTGGATCTGA
	Reverse	TTCCAGACCTCCCACCTCAA
	Probe	CTCTGCCTGCTGTTGCCAACATGC^*b*^
Primers used for RT-qPCR^*c*^		
GaHV-2-vTR	Forward	CCTAATCGGAGGTATTGATGGTACTG
	Reverse	CCCTAGCCCGCTGAAAGTC
	Probe	CCCTCCGCCCGCTGTTTACTCG^*b*^
GAPDH	Forward	GAAGCTTACTGGAATGGCTTTCC
	Reverse	GGCAGGTCAGGTGAACAACA
	Probe	TGTGCCAACCCCCAAT^*b*^
GaHV-2-ICP4	Forward	CGTGTTTTCCGGCATGTG
	Reverse	TCCCATACCAATCCTCATCCA
	Probe	CCCCCACCAGGTGCAGGCA^*b*^

a Mutations are shown in bold and are underlined.

b Modified with 5′-FAM and 3′-TAMRA (63). TAMRA, tetramethylrhodamine, a fluorescent label.

c vTR/chTR mismatches are underlined.

### Plaque size assay.

To assess the viral spread in cell culture, a plaque size assay was used as described previously (64). CEFs (10^6^) were infected with 100 plaque-forming units (PFU) of the corresponding revertant BAC constructs. After 6 days postinfection (dpi), viral growth was detected, and images of a minimum of 50 random plaques from each recombinant virus were taken. The plaque areas were measured using Image J software (NIH) and normalized to the wild-type virus. Three independent experiments were performed, and the difference in plaque areas was evaluated using ANOVA one-way analysis of variance.

### Multistep growth kinetics assay.

To further assess the replication properties of the recombinant viruses, a multistep growth kinetics assay was performed as described previously (64). CEFs (10^6^) were infected with 100 PFU of the corresponding recombinant BAC constructs. Cells were trypsinized and titrated on uninfected CEFs at specific time points. Three independent experiments were performed, and the difference in plaque areas was evaluated using ANOVA one-way analysis of variance.

### Reconstitution and propagation of GaHV-2 recombinant viruses.

Reconstitution of the viruses was done using ESCDL-1 cells line. Cells were transfected with the recombinant BAC constructs and cotransfected with a plasmid that encodes Cre-recombinase using a calcium phosphate transfection protocol as described previously (65). Following reconstitution, viruses were propagated in secondary CEFs. CEF cells were coinfected with ESCDL-1 cells containing reconstituted recombinant GaHV-2 virus, and viral titration was performed.

### Second animal experiment, cell isolation, DNA and RNA extraction.

White Leghorn specific pathogen-free B^13^B^13^ chickens, highly susceptible to GaHV-2, were obtained from INRA-Tours, France, and were used for the animal experiments. The animals were housed in isolated biosecurity level 3 facilities at Avian Virology and Immunology Service of Sciensano (Brussels, Belgium). Chickens were injected intramuscularly at the age of 2 days with 2,000 PFU of CEFs infected with either mutant (vTR-E2E3mut, *n* = 26) or revertant (vTR-E2E3rev, *n* = 26) recombinant virus. To assess viral loads and telomerase activity in infected animals, blood samples and feather follicle epitheliums (FFEs) were collected at 6, 13, 20, 28, 35, 41, 48, and 55 days postinfection (dpi) and weight progression was recorded at each time point. Animals were assessed daily for the onset of common Marek’s disease (MD) symptoms. At 55 dpi, chickens were euthanized and checked for tumor growth. PBLs from anticoagulated blood were isolated using Histopaque-1077 density gradient (Sigma-Aldrich) according to the manufacturer’s recommendations. Genomic DNA from each sample was isolated using the DNeasy blood and tissue kit (Qiagen) as described by the manufacturer. RNA from isolated PBLs was extracted using a guanidium thiocyanate-phenol-chloroform extraction (Tri-Reagent, Ambion).

### GaHV-2 viral loads during the course of infection.

GaHV-2 genome copies during viral infection were quantified using qPCR to determine the replication properties of the recombinant viruses. DNA (1 μg) extracted from blood collected from eight random animals in each group was used for qPCR analysis. Virus genome copies were assessed by qPCR with the No Rox Probe MasterMix dTTP (Takyon) using primers and probe specific for the major capsid protein VP5 of GaHV-2, according to the manufacturer’s recommendations. Primers used in qPCR assays are shown in Table 2. Virus genome copies were normalized against the chicken inducible nitric oxide synthase (iNOS) gene, as previously described (66). Briefly, for the generation of standard curves in qPCR assays, PCR products of the ICP4 or iNOS genes were used. Serial 10-fold dilutions of each target were used for generating standard curves, starting with approximately 100 ng of DNA. The standard curves were generated by plotting the cycle threshold (*C_T_*) value at each dilution with the total copy numbers.

### vTR expression in recombinant virus-infected cells.

vTR expression levels were determined *in vivo* by RT-qPCR from total RNA extracted from infected PBLs. DNase I (NEB) treatment was performed on all the samples, and cDNA was generated using the SuperScript IV (SSIV) reverse transcriptase (RT) (Invitrogen) according to the manufacturer’s recommendations. Following the RT, qPCR was performed to measure the expression of chicken TR (chTR) and vTR. The expression levels were normalized against the cellular GAPDH gene, as previously described (35). In addition, relative vTR expression was normalized to the expression of the viral gene coding for immediate early protein ICP4. Primers and probes used for RT-qPCR are shown in Table 2.

### Ethics statement.

The animal study was conducted following Belgian law for animal protection and the European Directive, 2010/63/EU. The ethics committee of Sciensano (file numbers LA1230174 [first experiment] and 20191016-03 [second experiment]) approved all animal experiments.
